# Supplementary material for: Complexome profiling of the Chlamydomonas psb28 mutant reveals TEF5 as an early PSII assembly factor
Source: Plant Cell. 2025 Mar 17;37(6):koaf055. doi: 10.1093/plcell/koaf055 (PMC12164590; doi:10.1093/plcell/koaf055)
Supplement: koaf055_Supplementary_Data [file koaf055_supplementary_data.zip › psb28_interactive.zip!/plots/intensity/qualityPlots/peptides/_Cre10.g447100.t1.2.html]

### Cre10.g447100.t1.2

Trivial name:   
SeqCoverage [median]: 20.60  
Mass: 27.32 kDa  
  
 

| Sequence | MissedCleavage | Mass [Da] | wtIntensity | muIntensity | Score |
| --- | --- | --- | --- | --- | --- |
| FLIYDKEQR | 1 | 1210.63 | 2228.32 | 6673.08 | 75.38 |
| GITAELGEYLR | 0 | 1220.64 | 2166.77 | 0.00 | 199.46 |
| KNYERPAQISGGPPAPFK | 2 | 1956.02 | 0.00 | 1470.81 | 39.00 |
| LTEAPGDTLLTLTR | 0 | 1499.82 | 3969.57 | 8602.69 | 134.13 |
| NYERPAQISGGPPAPFK | 1 | 1827.93 | 486.57 | 3133.79 | 105.39 |
|  |  |  | 8851.2 | 19880.4 |  |
